# T cell–dependent modulation of pain behaviors in a rat model of recurring paclitaxel-induced peripheral neuropathy

**DOI:** 10.1016/j.bbih.2026.101349

**Published:** 2026-09-08

**Authors:** Ahmed Olalekan Bakare, Paul J. Austin, Michele Curatolo, Kimberly E. Stephens, Eellan Sivanesan

**Affiliations:** a Department of Anesthesiology and Critical Care Medicine, Johns Hopkins University, School of Medicine, Baltimore, MD, USA; b School of Medical Sciences (Neuroscience), Brain and Mind Centre, University of Sydney, NSW, Australia; c Department of Anesthesiology & Pain Medicine, University of Washington, Seattle, WA, USA; d Department of Neuroscience, University of Arkansas for Medical Sciences, Little Rock, AR, USA

**Keywords:** Chemotherapy-induced peripheral neuropathy, Neuropathic pain, Paclitaxel, T cells, Chemotherapy-induced neuropathic pain

## Abstract

Advances in preventing or treating chemotherapy-induced peripheral neuropathy (CIPN) have been limited because most preclinical models do not capture the repeated cycles of chemotherapy exposure common in clinical practice. Consequently, the mechanisms driving CIPN across successive chemotherapy cycles remain incompletely understood. Here, we investigated the contribution of T cells to recurrent paclitaxel (PTX)-induced peripheral neuropathy (PIPN) using homozygous RNU rats (T cell–deficient) and heterozygous littermates (T cell–competent). PTX (8 mg/kg total) was administered intraperitoneally over two treatment cycles to model repeated chemotherapy exposure and its effects on neuropathic pain. Pain sensitivity testing with von Frey filaments, dry-ice, and acetone assays revealed distinct pain phenotypes across cycles. Mechanical allodynia was transient, resolving between cycles, with a smaller reduction in mechanical withdrawal threshold during the observed period following the second cycle, independent of T cell status. In contrast, cold hyperalgesia persisted in T cell-competent rats but did not reach significance in T cell–deficient rats, whereas cold allodynia was transient, resolving between cycles, and developed exclusively in T cell–competent rats. T cell frequencies in peripheral neural tissues were higher after the second PTX cycle than after the first, while natural killer cell populations showed T cell- and tissue-specific differences across cycles. CD163^+^ M2-like macrophage abundance remained relatively stable across cycles in peripheral neural tissues, whereas CD206^+^ M2-like and CD86^+^ M1 macrophage populations showed tissue-specific changes. Repeated exposure also reduced satellite glial cell gliosis and was associated with gene-specific transcriptional changes, including T cell–dependent effects linked to immune-glial signaling in the sciatic nerve. Together, these findings indicate that repeated paclitaxel exposure produces distinct transient and persistent pain phenotypes and engages tissue-specific neuroimmune mechanisms, with T cells selectively shaping sensory outcomes in PIPN.

## Highlights

• Repeated PTX produces distinct transient and persistent pain phenotypes.
• T cells shape cold pain behaviors but not mechanical allodynia resolution.
• T cell frequencies in DRG and sciatic nerve are higher after the second PTX cycle.
• Repeated PTX induces tissue-specific macrophage, B cell, and NK cell remodeling.
• Repeated PTX alters immune-glial signaling in the sciatic nerve.

## 1 Introduction

Substantial progress in cancer therapy over the past two decades has significantly improved patient survival. However, survival is frequently accompanied by chemotherapy-induced peripheral neuropathy (CIPN), a debilitating condition that currently lacks effective preventive or therapeutic strategies. CIPN is characterized by cold and mechanical allodynia, spontaneous pain, and affective disturbances, which may persist long after therapy cessation and severely impair quality of life (Flatters et al., 2017).

Paclitaxel (PTX), one of the most effective chemotherapeutic agents, is widely used to treat several malignancies, including non-small cell lung, breast, pancreatic, esophageal, ovarian, and prostate cancers (Baert et al., 2021; Sonabend et al., 2023). Despite its clinical efficacy, PTX frequently induces peripheral neuropathy through mechanisms that enhance neuronal and inflammatory signaling, including altered ion channel expression, pro-inflammatory cytokine release (TNF-α, IL-1β, and IL-6), and promote immune cell activation, including T cells, B cells, and macrophages, within the dorsal root ganglia (DRG) and peripheral nerves (Burgess et al., 2021; Makker et al., 2017). These neurotoxic and pathological processes lead to microtubule disruption, axonal degeneration, glial activation, and persistent neuroinflammation, which together increase nociceptor activation and drive pain hypersensitivity (da Costa et al., 2020; Gornstein and Schwarz, 2017). Clinically, neuropathic symptoms develop in up to 60% to 70% of patients receiving PTX (Flatters et al., 2017; Mo et al., 2022). Although some patients demonstrate partial recovery within approximately 3 months after treatment cessation (Flatters et al., 2017; Seretny et al., 2014), at least one-third experience incomplete resolution, with persistent symptoms lasting 6 months or longer (Burgess et al., 2021; Flatters et al., 2017; Seretny et al., 2014). In clinical practice, PTX is administered in repeated cyclic regimens (Baert et al., 2021; Mo et al., 2022), which increase cumulative drug exposure and consequently amplify systemic toxicity and neurotoxicity. Similarly, tumor recurrence after initial therapy is common across solid malignancies, often necessitating additional chemotherapy cycles (Nors et al., 2024; Pedersen et al., 2022; Stirling et al., 2021; Valdes et al., 2016). Re-treatment increases cumulative neurotoxic exposure, and a higher cumulative PTX dose has been associated with an increased risk of clinically significant PTX-induced peripheral neuropathy (PIPN) (da Costa et al., 2020). However, the biological mechanisms and phenotypic features of repeated or recurrent PIPN remain poorly defined.

Many preclinical PTX models use compressed dosing schedules with brief inter-treatment intervals. Typically, injections are administered over several consecutive days without the prolonged drug-free intervals and potential re-exposure observed clinically (Bacalhau et al., 2023; da Costa et al., 2020; Makker et al., 2017; Singh et al., 2022; Valdes et al., 2016). This divergence between clinical treatment patterns and preclinical modeling may limit translational relevance. Moreover, the mechanisms and pain phenotypes associated with recurrent PIPN remain poorly defined. Therefore, elucidating how peripheral sensory neurons and resident and infiltrating immune cells, including T cells, B cells, natural killer (NK) cells, and macrophages, respond to repeated PTX exposure is critical for the development of mechanism-based pain therapies.

Accumulating evidence implicates T cells as key regulators of peripheral neuroimmune interactions in neuropathic pain (Austin et al., 2012; Davoli-Ferreira et al., 2020; Fan et al., 2025; Kleinschnitz et al., 2006; Moalem et al., 2004; Singh et al., 2022). T cell infiltration into the peripheral nerves, DRG and spinal cord shape the development, maintenance, and resolution of pain through modulation of cytokine signaling and glial activation, including satellite glial, Schwann cells and astrocytes (Austin et al., 2012; Cao and DeLeo, 2008; Costigan et al., 2009; Kleinschnitz et al., 2006; Moalem et al., 2004). Reconstitution of T cells previously exposed to PTX accelerates PIPN recovery in mice, implicating adaptive immune memory and cytokine-mediated regulation of peripheral inflammation in pain resolution (Laumet et al., 2019; Singh et al., 2022). However, the role of T cells in the context of repeated PTX exposure and recurrent PIPN remains unclear. Moreover, many prior studies (Laumet et al., 2019; Singh et al., 2022) have relied on immunodeficient animal models that lack both T and B cells, limiting the ability to dissect the distinct contributions of individual lymphocyte subsets within an intact peripheral neuroimmune environment.

We hypothesized that T cells regulate recurrent PIPN by shaping macrophage phenotype, lymphocyte infiltration, and satellite glial and Schwann cell responses, thereby modulating neuroimmune signaling within peripheral sensory tissues. To test this hypothesis, we longitudinally quantified mechanical and cold hypersensitivity and assessed ongoing pain–like behavior, while comparing immune cell composition, macrophage polarization, and glial activation in T cell–competent and T cell–deficient rats subjected to recurrent PTX treatment. We used homozygous RNU^−/−^ rats, which are profoundly deficient in T cells but retain functional B and NK cells, and their heterozygous RNU ^±^ littermates as T cell-competent controls. The use of heterozygous littermates provides a genetically matched comparator while minimizing potential differences related to strain background (Rolstad, 2001; Sass et al., 1997).

## 2 Methods

### 2.1 Animals

We obtained homozygous (RNU^−/−^ #316; T cell–deficient) and heterozygous (RNU^+/−^ #118; T cell–competent) strains of adult male Rowett nude rats (Crl: NIH-Foxn1^rnu^) aged 8 weeks from Charles River Laboratories (Wilmington, MA, USA). Their average starting weight was 240 ± 17 g. Animals were housed in a controlled environment (temperature: 25 ± 1°C; humidity: 60 ± 10%) with a 12-h light/dark cycle and *ad libitum* access to food and water. Rats were acclimated to the facility for 7 days before any experimental procedures. They were randomly assigned to experimental groups, with each cage containing three rats from different treatment conditions. All behavioral assessments were conducted by an experimenter blinded to treatment allocation. All procedures were approved by the Johns Hopkins University Animal Care and Use Committee and were conducted in accordance with the NIH Guide for the Care and Use of Laboratory Animals. Group sizes were determined by power analysis (Sigma version 12.0; Systat Software, Inc., San Jose, CA), with a significance level of α = 0.05, power = 0.80, and effect sizes estimated from previous studies (Bakare et al., 2024, 2026; Sivanesan et al., 2019, 2023).

### 2.2 PTX administration

PTX (cat. #58055525; T7402, Millipore Sigma, Burlington, MA, USA) was administered according to previously established protocols (Sivanesan et al., 2023). Briefly, PTX was prepared by dissolving the compound in a 1:1 mixture of absolute ethanol and Cremophor EL (cat. #238470, Millipore Sigma), which was subsequently diluted with normal saline to yield a final concentration of 2 mg/mL. Rats received intraperitoneal (i.p.) injections of PTX (2 mg/kg) every other day. Each treatment cycle comprised four i.p. injections (totaling 8 mg/kg per cycle), and animals underwent two cycles of treatment.

### 2.3 Study design

**Fig. 1:**
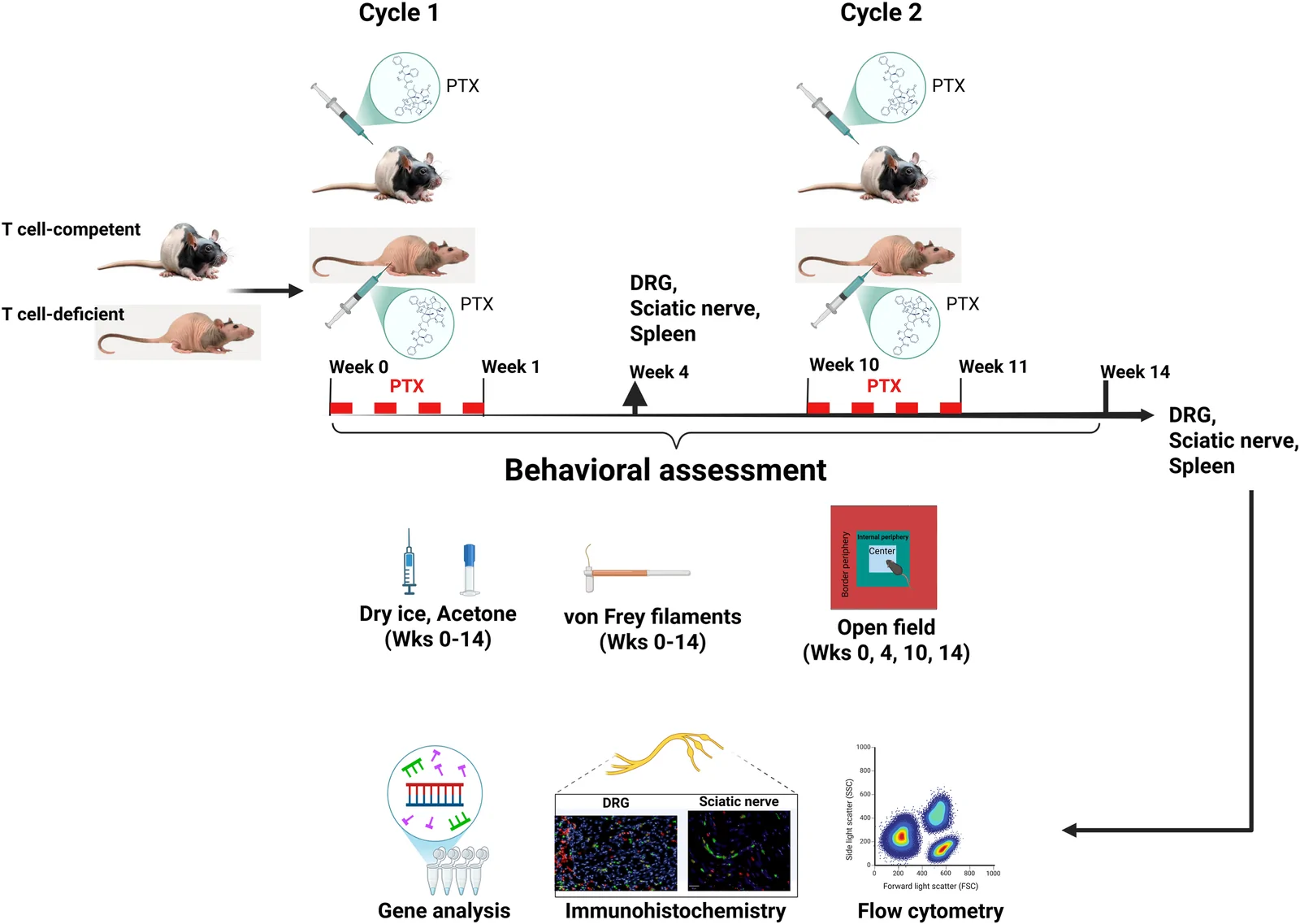
**Experimental protocol and timeline for recurrent PTX-induced peripheral neuropathy across two PTX treatment cycles.** T cell–competent (RNU^+/−^) and T cell–deficient (RNU^−/−^) rats underwent two cycles of PTX administration (2 mg/kg, intraperitoneal, every other day; red blocks). Pain-related behaviors were assessed weekly throughout the study, from baseline through post-treatment periods for both cycles. Reflexive and non-reflexive behavioral assays were used to evaluate neuropathic pain–related outcomes. Peripheral tissues, including DRG, sciatic nerve, and spleen, were collected at weeks 4 and 14 (3 weeks after completion of each PTX cycle) for immunohistochemistry, flow cytometry, and PCR analyses. DRG, dorsal root ganglia; PCR, polymerase chain reaction; PTX, paclitaxel. (For interpretation of the references to colour in this figure legend, the reader is referred to the Web version of this article.)

The study consisted of two experimental groups, each comprising 10 animals: PTX T cell–competent (PTX RNU ^±^ rats) and PTX T cell–deficient (PTX RNU^−/−^ rats; Fig. 1). Baseline behavior was assessed immediately after the acclimation period. Animals then received the first treatment cycle with PTX (week 0), as illustrated in Fig. 1. After the resolution of PTX-induced mechanical and cold allodynia (approximately week 10), a second cycle of PTX was administered at the same dosage as the first.

Comprehensive, behavioral assessments of mechanical allodynia, cold allodynia, and cold hyperalgesia, were conducted weekly. For the mechanical and cold sensitivity tests, both the right and left hind limbs were evaluated, and the mean of both sides was recorded as the final score. The open-field test was performed at baseline (week 0) and at weeks 4, 10, and 14.

A subset of animals from each group was euthanized 3 weeks after the first treatment cycle (week 4), whereas the remaining animals were euthanized 3 weeks after the second treatment cycle (week 14) under deep isoflurane anesthesia. The DRG, sciatic nerve, and spleen were collected for immunohistochemistry, flow cytometry, and gene expression analyses. All behavioral, biochemical, and data analyses were performed by investigators blinded to the treatment conditions. Biochemical assays were performed in duplicate to ensure reproducibility.

### 2.4 Mechanical allodynia test

Mechanical sensitivity was assessed by applying von Frey monofilaments (2–15 g; Stoelting Co., Wood Dale, IL, USA) with the up–down method as previously described (Dixon, 1980; Sivanesan et al., 2023). On test days, rats were acclimated for 30 min to individual Plexiglas chambers placed on an elevated mesh floor. Calibrated filaments were applied perpendicularly to the mid-plantar surface of the hind paw for approximately 5 s, beginning with a 2 g filament. A positive withdrawal response, defined as an abrupt paw withdrawal, sustained lifting, flicking, or licking, was recorded, and the next filament was selected according to the up–down algorithm. The withdrawal threshold was determined as the force (in grams) corresponding to the 50% withdrawal threshold.

### 2.5 Cold allodynia test

Cold allodynia was evaluated with the acetone spray method (Yoon et al., 1994). Rats were acclimated for 30 min to Plexiglas chambers on a mesh floor. A 100 μL aliquot of acetone was drawn into a 1 mL syringe and gently applied to the plantar surface of each hind paw through the mesh, avoiding direct skin contact. The frequency of paw flicking, licking, or scratching (behavioral responses) was recorded for 120 s after the application. Each paw was tested in three trials, separated by approximately 30-min intervals. The mean frequency of responses across trials from both limbs was calculated for each animal.

### 2.6 Cold hyperalgesia test

Cold hyperalgesia was evaluated by using the dry-ice method as previously described (Bakare et al., 2026; Brenner et al., 2012; Sivanesan et al., 2019). Rats were acclimated for 30 min to Plexiglass chambers positioned on a flat glass surface. Granulated dry ice was packed into a 12 mL syringe, and the tip of the syringe was gently applied to the underside of the glass directly beneath the plantar surface of the hind paw. The latency to paw withdrawal (paw withdrawal time in seconds) was recorded as a measure of cold sensitivity. To prevent tissue injury, a cutoff time of 45 s was applied. Each paw was tested in three trials separated by 15-min intervals, and the mean withdrawal latency was calculated for analysis.

### 2.7 Open-field test

Non-reflexive pain–related behavior, exploratory activity, and anxiety-like responses were evaluated using the open-field test as described previously (Sivanesan et al., 2023). The same animals were assessed longitudinally at baseline and at weeks 4, 10, and 14. Rats were acclimated to the testing environment for 1 h before being assessed. Each animal was placed individually in a plastic box (73 × 45 × 35 cm) with the floor conceptually divided into three zones: border periphery, internal periphery, and center (Fig. 1). The locomotor activity of each rat was recorded for 10 min by a high-resolution camera and analyzed with SMART 3.0 software (Panlab, Harvard Apparatus, Barcelona, Spain). The arena was cleaned with 70% ethanol and dried between test sessions to eliminate residual scent cues.

Details of flow cytometry (Supplementary Fig. 1), immunohistochemistry, RNA isolation, and quantitative real-time PCR (qPCR) analysis can be found in the supplementary methods.

### 2.8 Statistical analysis

All data are presented as mean + standard error of the mean and were analyzed with GraphPad Prism version 10.4.0 (GraphPad Software, La Jolla, CA, USA). Behavioral data were analyzed by two-way repeated-measures ANOVA, with time as the within-subject factor and T cell status as the between-subject factor, followed by Tukey's multiple-comparisons test. Relative gene expression, immunohistochemistry, and flow cytometry data were analyzed by one-way ANOVA followed by Tukey's multiple-comparisons test, or by Student's *t*-test, as appropriate. Statistical significance was set at *p* < 0.05.

## 3 Results

### 3.1 Resolution of PTX-induced mechanical allodynia between cycles occurs independently of T cells

**Fig. 2:**
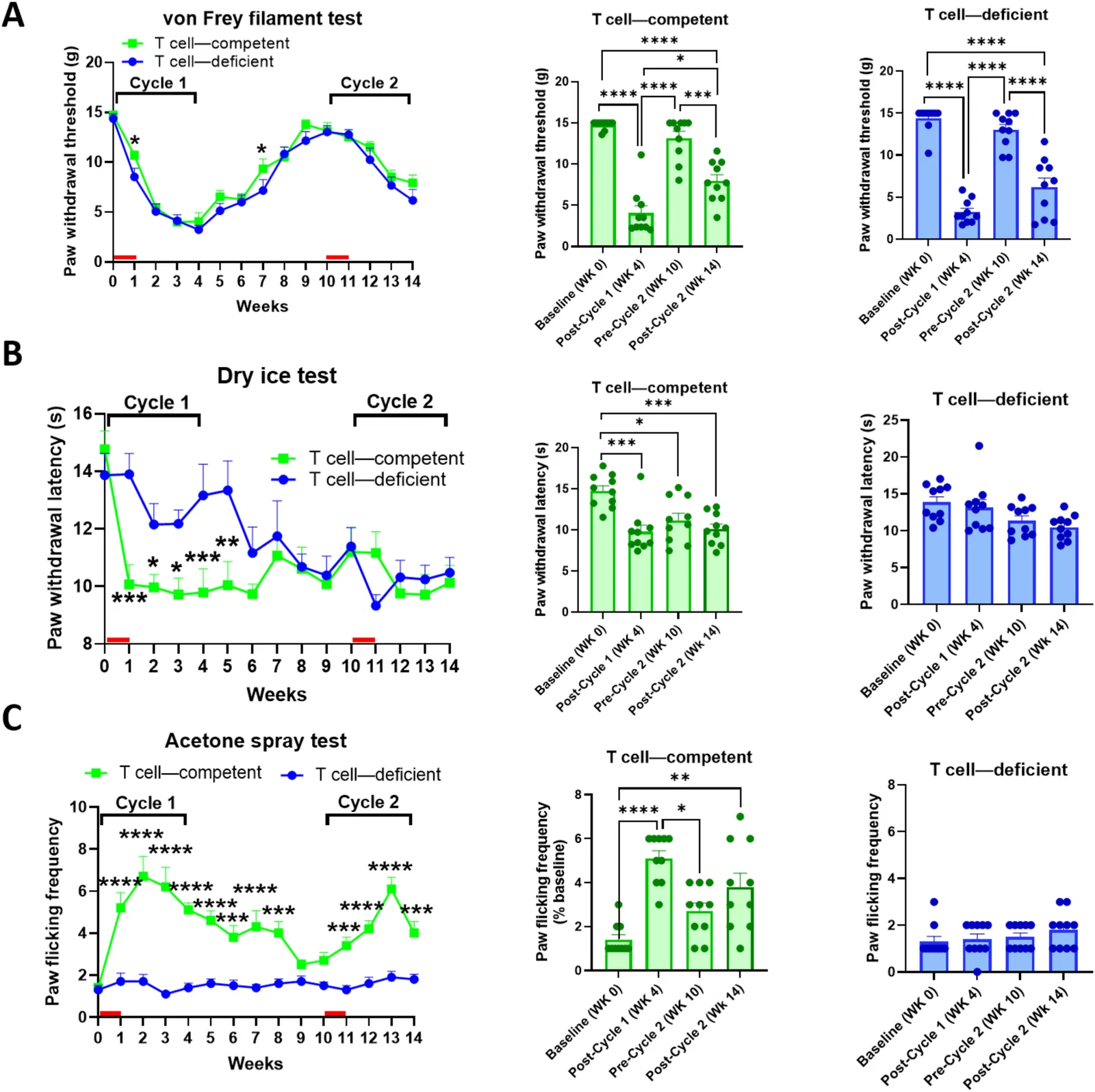
**T cells differentially regulate mechanical and cold hypersensitivity across two PTX treatment cycles. (A)** During the observed post-treatment period, mechanical allodynia was less pronounced after the second PTX cycle than after the first and resolved between cycles in both T cell–competent and T cell–deficient rats. **(B)** Cold hyperalgesia persisted across treatment cycles in T cell-competent rats, whereas T cell-deficient rats did not develop significant cold hyperalgesia relative to baseline. **(C)** Cold allodynia developed exclusively in T cell–competent rats and resolved between treatment cycles. PTX was administered during weeks 0 and 10 (red bars). Data are presented as mean + SEM. Behavioral data were analyzed by two-way repeated-measures ANOVA with time as the within-subject factor and T cell status as the between-subject factor, followed by Tukey's multiple-comparisons test. Comparisons shown in the summary bar graphs were derived from the same repeated-measures analysis. *p < 0.05, **p < 0.01, ***p < 0.001, ****p < 0.0001. N = 10 per group. PTX, paclitaxel; SEM, standard error of the mean. (For interpretation of the references to colour in this figure legend, the reader is referred to the Web version of this article.)

PTX decreased mechanical withdrawal thresholds to von Frey filaments after each treatment cycle in both T cell–deficient and T cell–competent rats. After the first PTX cycle, mechanical thresholds declined in both groups, with a small, transient difference at week 1 between T cell–competent and -deficient rats (Fig. 2A). However, thresholds were similar between groups from week 2 onward. Mechanical allodynia gradually resolved in both groups, with partial recovery by week 7 and near-complete resolution by week 9, when thresholds approached baseline levels. During the observed period after the second PTX treatment cycle, the decline in mechanical thresholds was smaller than that observed after the first cycle in both groups. Consistent with this finding, mechanical thresholds measured 3 weeks after the second PTX cycle (week 14) remained higher than those measured 3 weeks after the first cycle (week 4) in T cell–competent rats, but this difference was not significant in T cell–deficient rats (Fig. 2A). Because mechanical thresholds had not returned to baseline by the end of the observation period, these findings do not distinguish a reduction in the magnitude of mechanical hypersensitivity from a change in its time course. Overall, the mechanical response to PTX was largely similar between T cell-competent and T cell-deficient rats. Overall behavioral findings are summarized in Supplementary Table 3.

### 3.2 PTX-induced cold hyperalgesia persists across treatment cycles without recovery in T cell–competent rats

Cold sensitivity assessed with the dry-ice test showed that T cell–competent rats developed persistent cold hyperalgesia after the first PTX treatment cycle, reflected by reduced hind paw withdrawal latency to cold (Fig. 2B). In these rats, cold hyperalgesia emerged rapidly, beginning at week 1 after PTX administration and persisting throughout the experimental period. In contrast, T cell–deficient rats did not develop significant cold hyperalgesia relative to baseline, although withdrawal latencies gradually decreased over the course of the experiment and approached those of T cell-competent rats at later time points. Notably, T cell–competent rats did not show evidence of recovery prior to initiation of the second PTX treatment cycle (week 10). Likewise, cold hyperalgesia did not worsen following the second PTX treatment cycle, as withdrawal latencies remained similarly reduced across both treatment periods in T cell–competent rats (Fig. 2B). Together, these findings indicate that PTX-induced cold hyperalgesia persisted across treatment cycles in T cell–competent rats, whereas T cell-deficient rats did not develop significant cold hyperalgesia relative to baseline.

### 3.3 Cold allodynia develops in PTX-treated T cell–competent but not T cell–deficient rats

Cold allodynia assessed with the acetone test showed that PTX treatment increased paw flicking/licking responses in T cell–competent rats but not in T cell–deficient rats (Fig. 2C). In T cell–competent rats, cold allodynia emerged rapidly after the first PTX treatment cycle, with increased paw flicking/licking responses evident by week 1 and resolving by week 9, when responses returned to baseline levels. After the second PTX treatment cycle, cold allodynia again developed only in T cell–competent rats, but with a slower onset than after the first cycle. Paw flicking/licking responses 3 weeks after the second PTX cycle (week 14) were comparable to those observed 3 weeks after the first cycle (week 4; Fig. 2C). Together, these findings demonstrate a T cell-dependent pattern of PTX-induced cold allodynia across both treatment cycles.

### 3.4 T cells are associated with reduced anxiety-related exploratory behavior after the second PTX treatment cycle

**Fig. 3:**
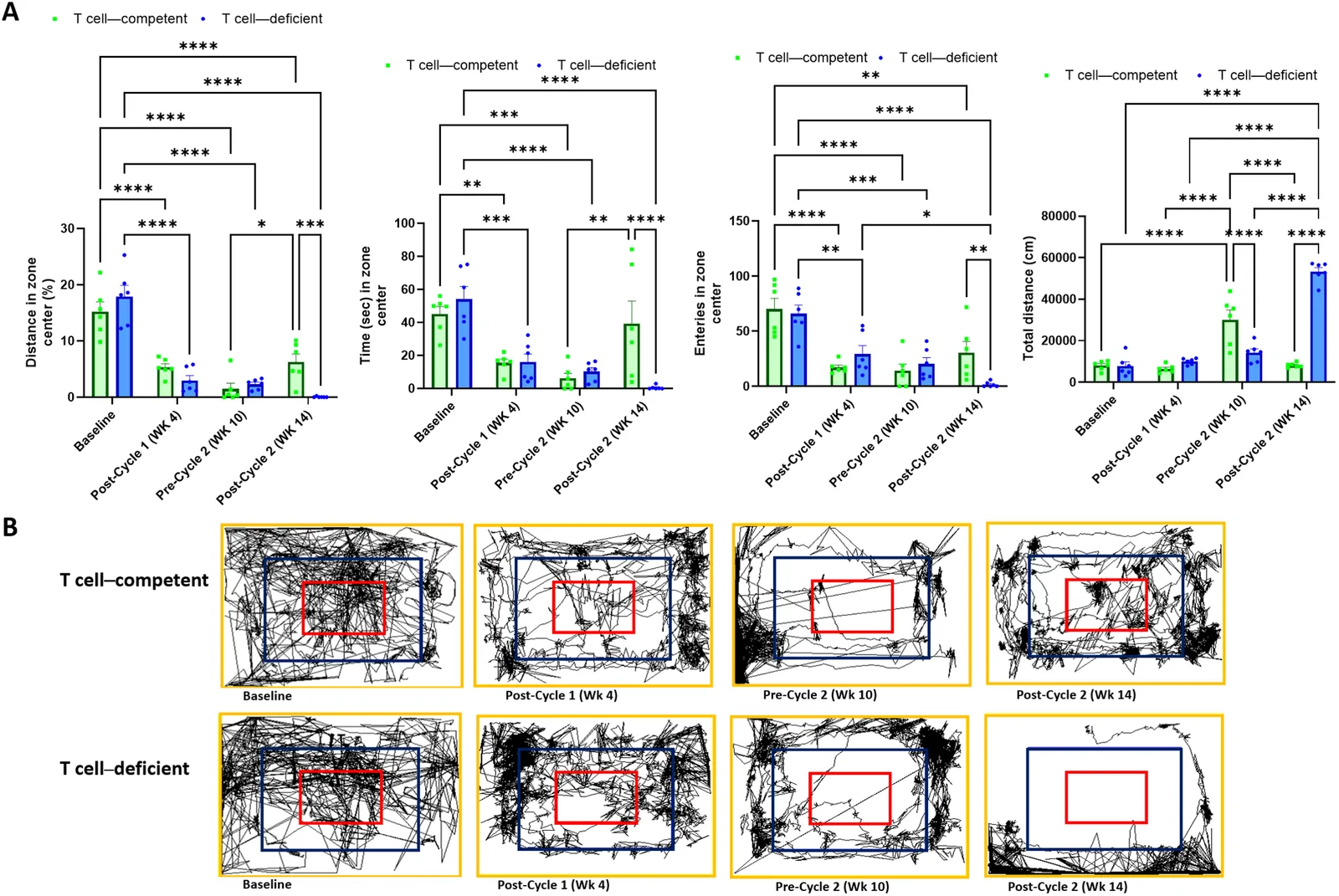
**T cell status is associated with differences in anxiety-related exploratory behavior after repeated paclitaxel exposure. (A)** PTX treatment was associated with changes in open-field exploratory behavior. After the second PTX cycle, T cell–competent rats exhibited greater center-zone exploratory activity than T cell–deficient rats, including greater distance traveled and time spent in the center zone. **(B)** Representative automated open-field track paths from T cell–competent and T cell–deficient rats. The same animals were assessed longitudinally at baseline and at weeks 4, 10, and 14. Red and yellow boxes denote the boundaries of the central and peripheral border zones, respectively. Data are presented as mean + SEM. Two-way repeated-measures ANOVA with time as the within-subject factor and T cell status as the between-subject factor, followed by Tukey's multiple-comparisons test. *p < 0.05, **p < 0.01, ****p < 0.0001. N = 6 per group. PTX, paclitaxel; SEM, standard error of the mean. (For interpretation of the references to colour in this figure legend, the reader is referred to the Web version of this article.)

We assessed anxiety-like behavior in the open-field test by measuring distance traveled in the center zone, time spent in the center, and center-zone entries. At baseline, T cell-competent and T cell-deficient rats showed similar center-zone exploratory behavior (Fig. 3A). Following the first PTX cycle (week 4) and before the second cycle (week 10), center-zone exploration decreased in both groups relative to baseline. Distance traveled in the center zone was reduced in T cell–competent rats at week 10 relative to week 4, although the contribution of repeated exposure to the open-field environment to this longitudinal change cannot be excluded.After the second PTX cycle (week 14), T cell–deficient rats displayed less center-zone exploration than T cell–competent rats, reflected by reduced center-zone distance traveled, time spent in the center, and center-zone entries. T cell–competent rats also showed increased center-zone exploratory behavior at week 14 relative to week 10, with increased distance traveled and time in the center zone (Fig. 3A and B).

Total distance traveled increased in T cell–deficient rats after the second PTX cycle despite reduced center-zone exploration, indicating that the reduction in center-zone exploration was not explained by reduced overall locomotor activity. Together, these findings demonstrate differences in anxiety-related exploratory behavior between T cell-competent and T cell-deficient rats following repeated PTX exposure.

### 3.5 Macrophage populations show tissue-specific and T cell-dependent changes across PTX treatment cycles

**Fig. 4:**
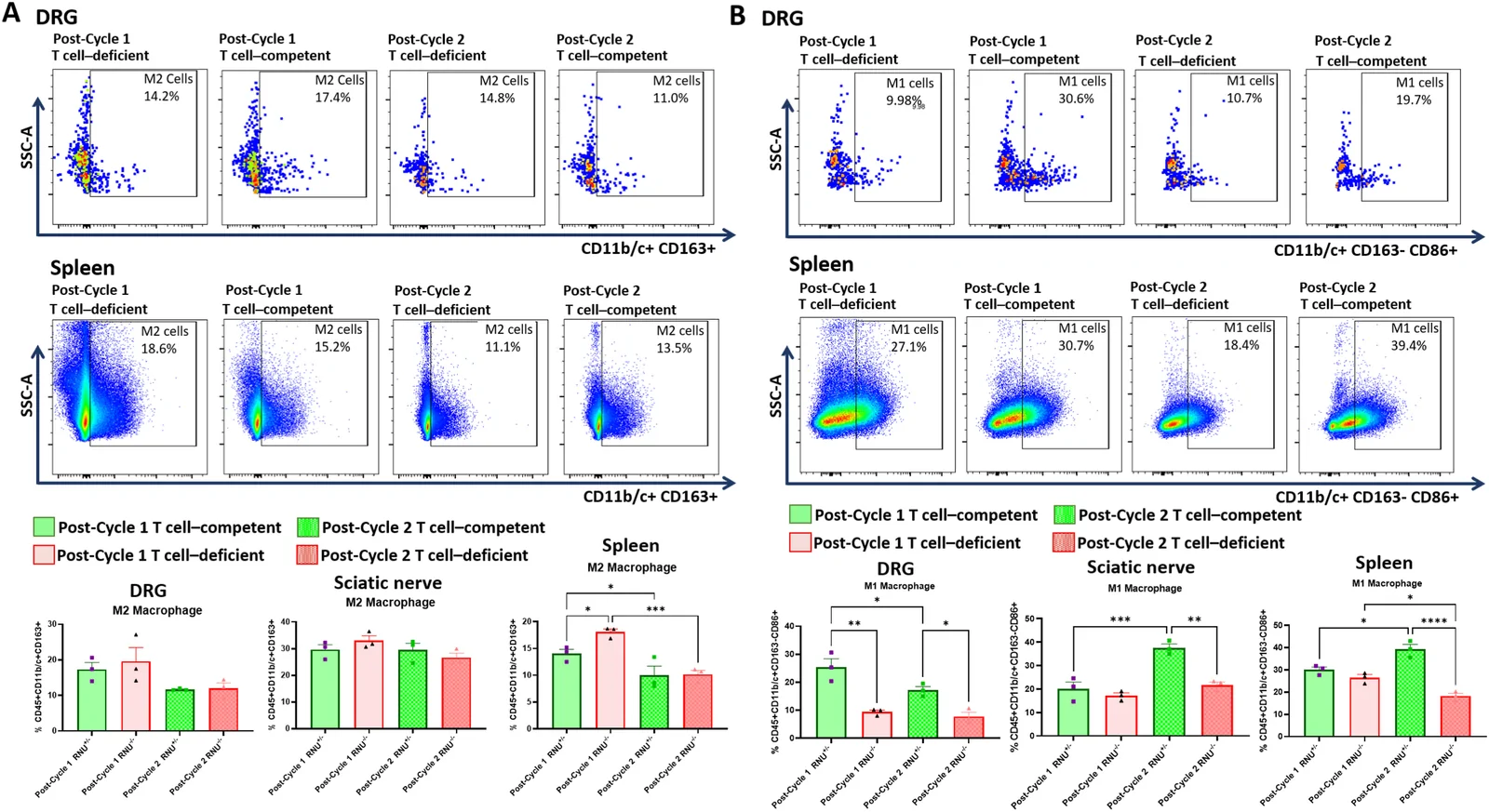
**Macrophage populations show tissue-specific and T cell-dependent differences across PTX treatment cycles. (A)** Representative flow cytometry plots of M2 macrophages (live CD45^+^CD11b/c^+^CD163^+^) in the DRG and spleen. After both the first and second PTX cycles, M2 macrophage frequencies in the DRG and sciatic nerve did not differ between T cell–competent and T cell–deficient rats. In contrast, splenic M2 macrophage frequency was reduced after the second PTX cycle compared with the first, independent of T cell status. **(B)** Representative plots of CD86^+^ M1 macrophages (live CD45^+^CD11b/c^+^CD163^−^CD86^+^) in the DRG and spleen. After the second PTX cycle, M1 macrophage frequencies were reduced in the DRG of T cell–competent rats and spleen of T cell–deficient rats, whereas M1 macrophages were increased in the sciatic nerve and spleen of T cell–competent but not T cell–deficient rats. M2 macrophage populations were quantified within the total live CD45^+^CD11b/c^+^ myeloid cell population for each tissue, while M1 macrophage populations were quantified within the total live CD45^+^CD11b/c^+^CD163^-^ myeloid cell population. Data are presented as mean + SEM. One-way ANOVA with Tukey's post hoc test. *p < 0.05, **p < 0.01, ***p < 0.001, ****p < 0.0001. N = 3 per group. M, macrophage; PTX, paclitaxel; RNU^+/−^, T cell–competent; RNU^−/−^, T cell–deficient; SEM, standard error of the mean.

We have previously shown that PTX decreases M2-like macrophages (CD11b/c^+^CD163^+^) irrespective of T-cell status compared with naïve rats (Bakare et al., 2026). In the present study, flow cytometry showed that CD163^+^ M2-like macrophage frequencies in the DRG and sciatic nerve were comparable between T cell–competent and T cell–deficient rats after both PTX treatment cycles (Fig. 4A). In contrast, splenic CD163^+^ macrophages were reduced after the second PTX cycle in both groups, consistent with a T cell–independent effect in the systemic compartment. A modest strain difference was observed after the first cycle, with lower splenic CD163^+^ macrophage frequencies in T cell–competent rats. A summary of flow cytometric immune cell changes across tissues and treatment cycles is provided in Supplementary Table 4.

Immunohistochemistry revealed tissue-specific changes in M2-like macrophage subpopulations. CD68^+^CD206^+^ M2-like macrophages increased in the DRG of both strains after the second PTX cycle (Supplementary Fig. 2A). In the sciatic nerve, CD68^+^CD206^+^ M2-like macrophages increased only in T cell–deficient rats (Supplementary Fig. 2B). These findings indicate shifts in M2-like macrophage subpopulations despite similar overall CD163^+^ M2-like macrophage frequencies.

**Fig. 5:**
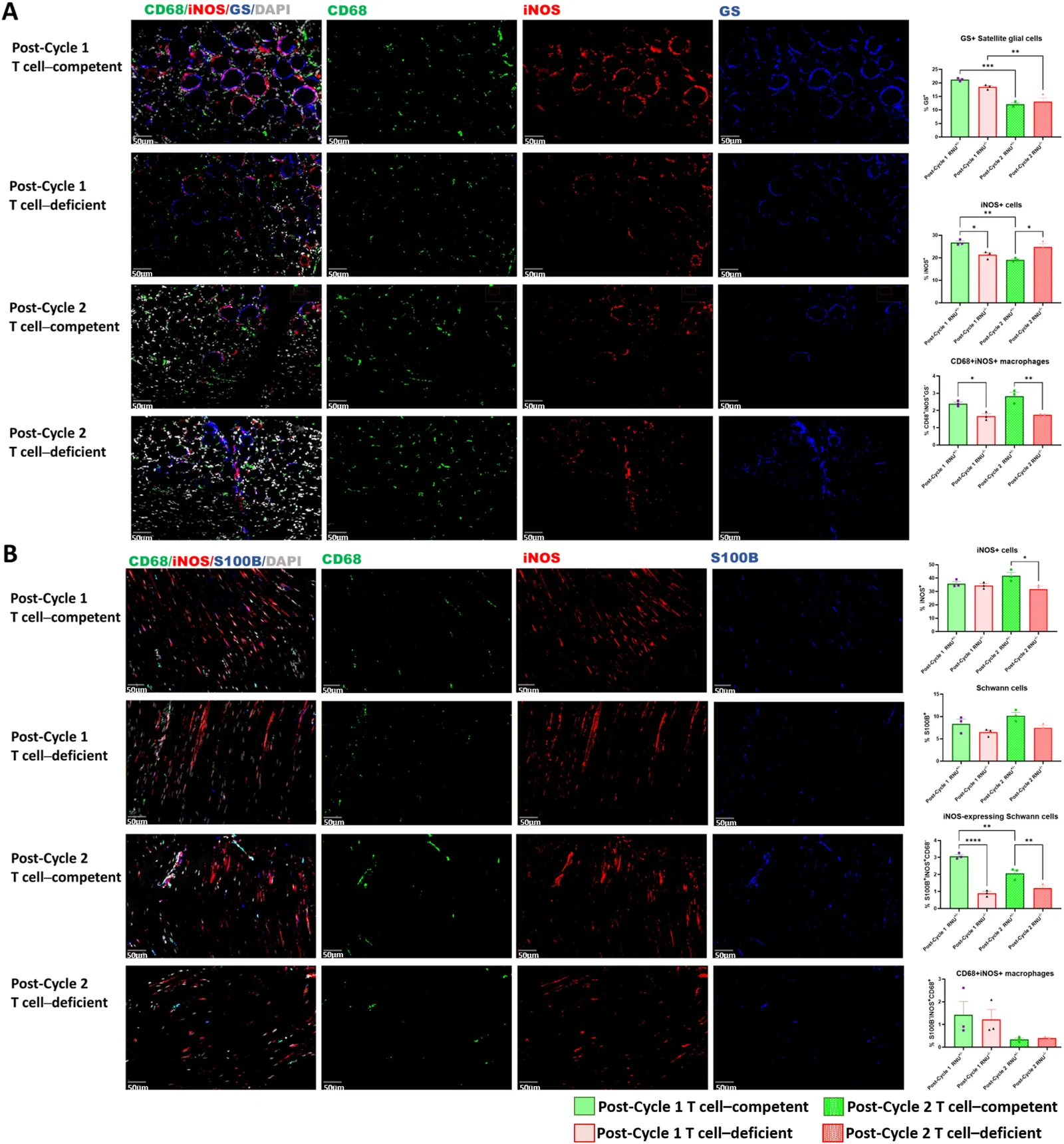
**iNOS** + **cells in the DRG and S100B** + **iNOS** + **Schwann cells in the sciatic nerve differ across PTX treatment cycles in a T cell-dependent manner. (A)** Representative confocal images and quantification of satellite glial cells, iNOS^+^ cells, and CD68^+^iNOS^+^ macrophages in the DRG of T cell–deficient and T cell–competent rats. Total iNOS^+^ cell frequencies were reduced after the second PTX cycle in T cell–competent but not T cell–deficient rats; CD68^+^iNOS^+^ macrophages did not differ between cycles but exhibited strain differences at both time points. Satellite glial cell gliosis was reduced after the second cycle in both genotypes. **(B)** Representative images and quantification of iNOS^+^ cells, Schwann cells, iNOS-expressing Schwann cells, and CD68^+^iNOS^+^ macrophages in sciatic nerve. iNOS-expressing Schwann cells (S100B^+^iNOS^+^) were reduced after the second PTX cycle in T cell–competent but not T cell–deficient rats, whereas total iNOS^+^ cell, CD68^+^iNOS^+^ macrophage, and Schwann cell abundance did not differ between cycles. Data are presented as mean + SEM. One-way ANOVA with Tukey's post hoc test *p < 0.05, **p < 0.01, ***p < 0.001, ****p < 0.0001. N = 3 per group. DRG, dorsal root ganglia; GS, glutamine synthetase; iNOS, inducible nitric oxide synthase; PTX, paclitaxel; RNU^+/−^, T cell–competent; RNU^−/−^, T cell–deficient; SEM, standard error of the mean.

In contrast, proinflammatory CD86^+^ M1-like macrophages (CD11b/c^+^CD163^−^CD86^+^) were differentially regulated in a tissue- and T cell–dependent manner across neural and systemic compartments (Fig. 4B). In the DRG of T cell–competent rats and spleen of T cell–deficient rats, CD86^+^ macrophages were reduced after the second PTX cycle. However, in the sciatic nerve and spleen, CD86^+^ macrophage frequencies were higher after the second than after the first PTX cycle in T cell–competent rats. T cell–deficient rats exhibited lower CD86^+^ macrophage frequencies than T cell–competent rats in the DRG after both treatment cycles. After the second cycle, CD86^+^ macrophage frequencies were also higher in the sciatic nerve and spleen of T cell–competent rats than in T cell–deficient rats. Thus, repeated PTX exposure was associated with increased sciatic nerve and splenic CD86^+^ macrophage frequencies only in T cell–competent rats. Immunohistochemical assessment further showed strain-dependent differences in CD68^+^ inducible nitric oxide synthase-positive (iNOS^+^) macrophage populations in the DRG, but not in the sciatic nerve (Fig. 5). CD68^+^iNOS^+^ macrophage frequencies did not differ between PTX treatment cycles in either tissue.

These results demonstrate tissue-specific differences in M2-like and CD86^+^ macrophage populations across PTX treatment cycles, with several changes differing according to T cell status.

### 3.6 B cell populations differ across PTX treatment cycles in a tissue-specific and T cell–dependent manner

**Fig. 6:**
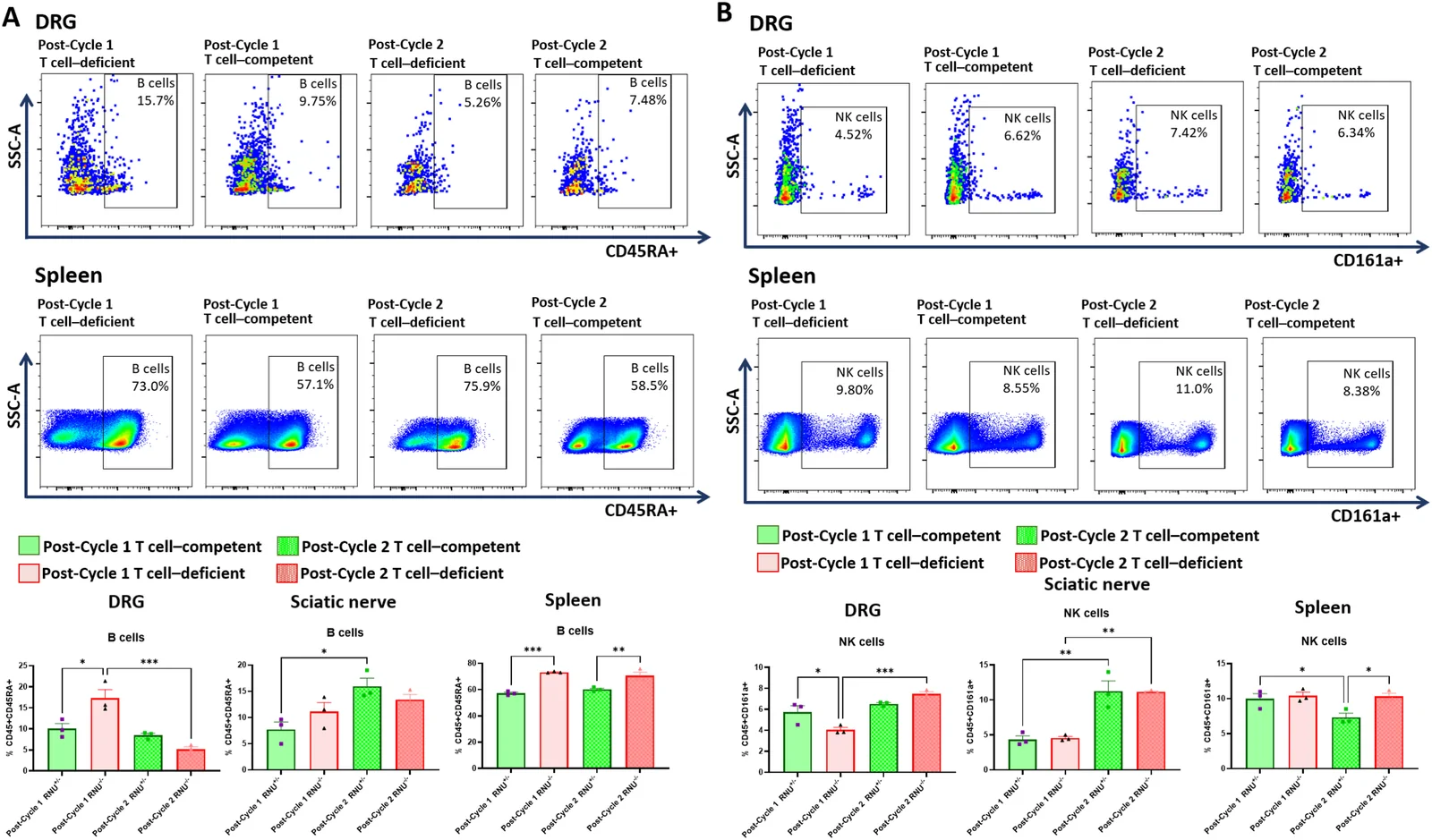
**B and NK cell populations differ across PTX treatment cycles in a tissue- and T cell-dependent manner. (A)** Representative flow cytometry plots of B cells (live CD45^+^CD45RA^+^) in the DRG and spleen. After the second PTX cycle, B cell frequency was reduced in the DRG of T cell–deficient rats, increased in the sciatic nerve of T cell–competent rats, and unchanged in the spleen. **(B)** Representative flow cytometry plots of NK cells (live CD45^+^CD161a^+^) in the DRG and spleen. After the second PTX cycle, NK cell frequency was increased in the DRG of T cell–deficient rats and in the sciatic nerve independent of T cell status, and unchanged in the spleen. B and NK cell populations were quantified within the total live CD45^+^ cell population in the DRG and sciatic nerve and total live CD45^+^ lymphocyte cells in the spleen. Data are presented as mean + SEM. One-way ANOVA with Tukey's post hoc test. *p < 0.05, **p < 0.01, ***p < 0.001. N = 3 per group. DRG, dorsal root ganglia; NK, natural killer; PTX, paclitaxel; RNU^+/−^, T cell–competent; RNU^−/−^, T cell–deficient; SEM, standard error of the mean.

We have previously shown that PTX decreases B cells (CD45^+^CD45RA^+^) in the DRG of both T-cell–competent and T-cell–deficient rats, whereas in the sciatic nerve, this decrease was observed only in T-cell–competent rats, compared with naïve rats (Bakare et al., 2026). In the current study, flow cytometric analysis revealed tissue-specific differences in B cell frequencies (CD45^+^CD45RA^+^) across PTX treatment cycles (Fig. 6A). After the first PTX treatment cycle, B cell frequencies in the DRG were higher in T cell–deficient rats than in T cell–competent rats. After the second PTX cycle, B cell levels in the DRG were comparable between strains, reflecting a reduction in B cell abundance in the ganglia of T cell–deficient rats relative to the first cycle. In contrast, B cell frequencies in the sciatic nerve increased between treatment cycles selectively in T cell–competent rats. Splenic B cell frequencies were consistently higher in T cell–deficient rats across both treatment cycles, with no appreciable change between cycles. Collectively, these findings demonstrate tissue-specific differences in B cell populations across PTX treatment cycles, with several changes dependent on T cell status.

### 3.7 NK cell populations differ across PTX treatment cycles in a tissue-specific and T cell–dependent manner

PTX decreases NK cells (CD45^+^CD161a^+^) in the peripheral nerve of T-cell–deficient rats but not T-cell–competent rats, compared with naïve rats (Bakare et al., 2026). Flow cytometric analysis in the current study revealed tissue-specific changes in NK cell frequencies (CD45^+^CD161a^+^) across PTX treatment cycles (Fig. 6B). In the DRG, NK cell frequencies increased in T cell–deficient rats after the second PTX treatment cycle compared with the first cycle, whereas NK cell levels in T cell–competent rats remained relatively stable across cycles. Accordingly, NK cell frequencies in the DRG were lower in T cell–deficient rats than in T cell–competent rats after the first cycle but were comparable between strains after the second cycle.

In the sciatic nerve, NK cell frequencies increased between PTX cycles in both T cell–competent and T cell–deficient rats, with no differences observed between strains. In contrast, splenic NK cell frequencies decreased in T cell–competent rats after the second PTX cycle relative to the first cycle.

Further characterization of NK cell subpopulations revealed increases in both mature (CD45^+^CD161a^+^CD11b/c^+^) and immature (CD45^+^CD161a^+^CD11b/c^−^) NK cells in the DRG after the second PTX cycle, independent of T cell status (Supplementary Fig. 3A). In the sciatic nerve, mature NK cells increased after the second PTX cycle in both strains, whereas immature NK cells increased selectively in T cell–competent rats. In the spleen, mature NK cell frequencies remained largely unchanged between PTX cycles but were consistently lower in T cell–competent rats, whereas immature NK cells were higher in T cell–competent rats after the first cycle (Supplementary Fig. 3B).

These findings demonstrate tissue-specific differences in NK cell populations across PTX treatment cycles, with T cell–dependent differences in overall NK cell frequencies in the DRG and spleen and differences in NK cell maturation states across cycles.

### 3.8 T cell frequencies and CD4/CD8 balance differ across PTX treatment cycles in a tissue-specific manner

**Fig. 7:**
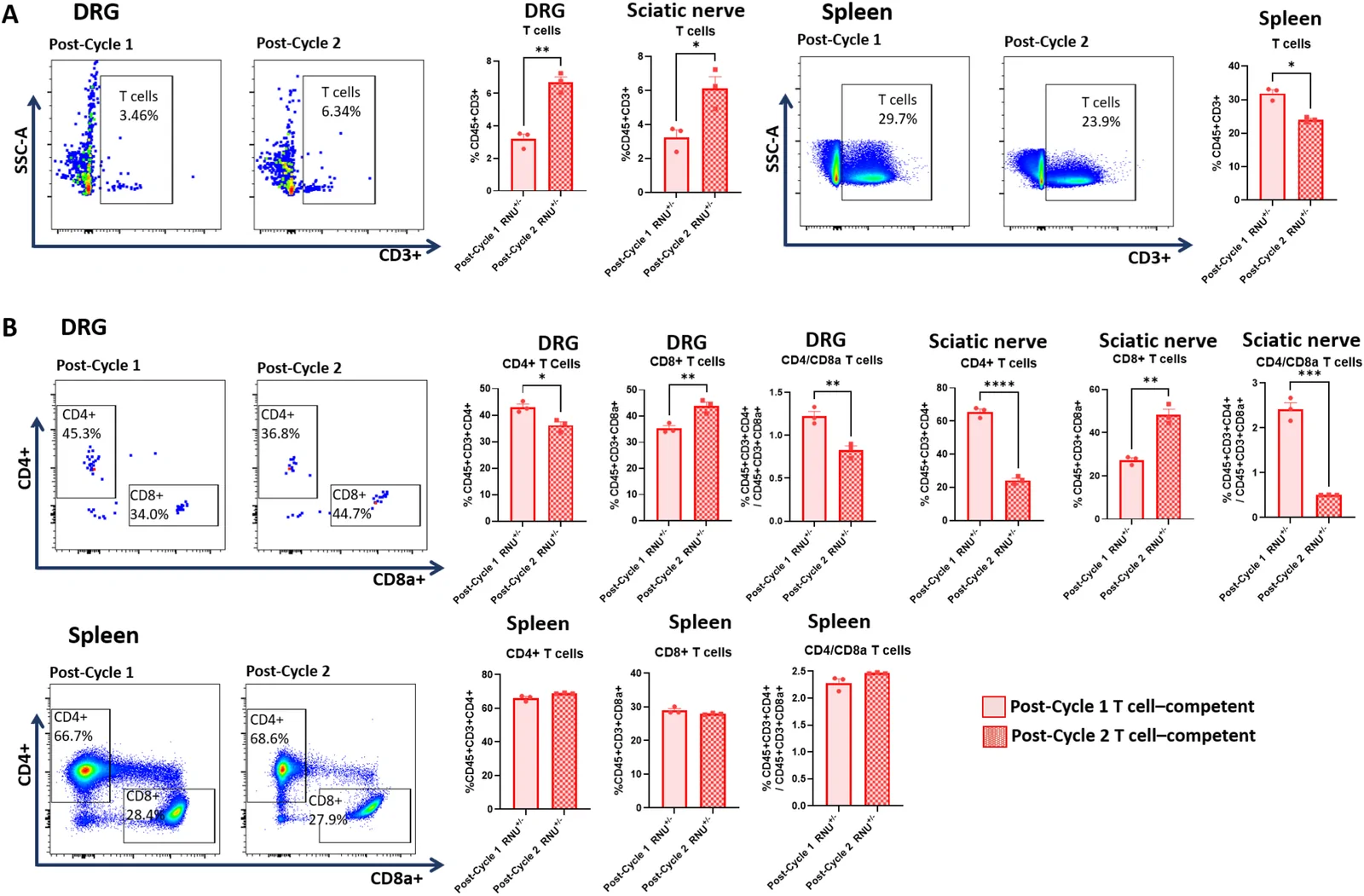
**Neural and systemic T cell frequencies and CD4/CD8 balance differ across PTX treatment cycles. (A)** Representative flow cytometry plots and quantification of total T cells (live CD45^+^CD3^+^) in DRG and spleen. T cell frequencies were higher in the DRG and sciatic nerve and lower in the spleen after the second PTX cycle than after the first. T cells were quantified within the total live CD45^+^ population in DRG and sciatic nerve and within the live CD45^+^ lymphocyte population in spleen. **(B)** Representative plots and quantification of CD4^+^ and single-positive CD8^+^ T cell subsets in DRG and spleen. After the second PTX cycle, CD4^+^ T cell frequencies were lower and CD8^+^ T cell frequencies were higher after the second cycle in the DRG and sciatic nerve, whereas frequencies were unchanged in the spleen. The CD4/CD8 ratio was decreased in the DRG and sciatic nerve, and unchanged in the spleen. CD4^+^ and single-positive CD8^+^ (CD4^−^CD8a^+^) T cells were quantified within the live CD45^+^CD3^+^ population in each tissue, with CD4^+^CD8a^+^ double-positive cells excluded from the CD8^+^ population. Data are presented as mean + SEM. Statistical analysis was performed by using unpaired Student's *t*-tests. *p < 0.05, **p < 0.01, ***p < 0.001, ****p < 0.0001. N = 3 per group. DRG, dorsal root ganglia; PTX, paclitaxel; RNU^+/−^, T cell–competent; RNU^−/−^, T cell–deficient; SEM, standard error of the mean.

PTX decreases T cells (CD45^+^CD3^+^) in peripheral nerve tissue of T-cell–competent rats compared with naïve rats (Bakare et al., 2026). Flow cytometric analysis in the study revealed tissue-specific differences in T cell frequencies (CD45^+^CD3^+^) across PTX treatment cycles (Fig. 7A). Specifically, T cell frequencies increased in both the DRG and sciatic nerve after the second PTX cycle compared with the first cycle. In contrast, splenic T cell frequencies decreased after the second PTX cycle compared with the first. Further analysis of T cell subsets showed that helper CD4^+^ and single-positive CD8^+^ (CD45^+^CD3^+^CD4^−^CD8a^+^) T cell frequencies remained largely unchanged in the spleen across treatment cycles (Fig. 7B). In the DRG and sciatic nerve, however, CD4^+^ T cell frequencies decreased while single-positive CD8^+^ T cell frequencies increased after the second PTX cycle. Consistent with these findings, the CD4^+^/CD8^+^ T cell ratio decreased in the DRG and sciatic nerve, while remaining unchanged in the spleen.

### 3.9 iNOS ^+^ cells in the DRG and iNOS ^+^ Schwann cells in the sciatic nerve differ across PTX treatment cycles

Assessment of satellite glial cell gliosis after repeated PTX exposure revealed changes in glutamine synthetase positive (GS^+^) cells in DRG (Fig. 5A). GS^+^ cells decreased after the second PTX treatment cycle compared with the first cycle in both T cell–competent and T cell–deficient rats, with no differences between strains within each cycle. In contrast, iNOS^+^ cell frequencies in the DRG differed by strain and treatment cycle. Total iNOS^+^ cell counts were higher in T cell–competent rats than in T cell–deficient rats after the first PTX cycle. After the second cycle, total iNOS^+^ cell frequencies decreased in T cell–competent rats but not in T cell–deficient rats, shifting the direction of the strain difference observed after the first cycle.

In the sciatic nerve, the abundance of S100B^+^ Schwann cells did not differ between treatment cycles or between strains (Fig. 5B). Total iNOS^+^ cell frequencies were similar between strains after the first PTX cycle but were higher in T cell–competent rats than in T cell–deficient rats after the second cycle. Notably, iNOS^+^ Schwann cells (S100B^+^iNOS^+^) were more abundant in T cell–competent rats across both cycles. iNOS^+^ Schwann cell numbers decreased after the second cycle in T cell–competent rats but not in T cell–deficient rats.

These findings demonstrate lower DRG GS^+^ cell abundance after the second PTX cycle than after the first, independent of T cells. In T cell–competent rats, iNOS^+^ cell frequencies in the DRG and iNOS^+^ Schwann cell frequencies in the sciatic nerve were also lower after the second cycle, whereas total iNOS^+^ cell frequencies in the sciatic nerve were not reduced.

### 3.10 Transcriptional programs in the sciatic nerve differ across PTX treatment cycles in a T cell–dependent and -independent manner

To determine whether the T cell–dependent cellular changes observed in the sciatic nerve were accompanied by differences in transcriptional programs across PTX treatment cycles, we performed qPCR analysis after repeated PTX exposure. qPCR analysis revealed differences in the expression of genes associated with inflammatory signaling and Schwann cell–related neuroimmune communication, with gene-specific dependence on T cell status (Supplementary Fig. 4).

Analysis of genes associated with Schwann cell function and neuroinflammatory signaling (Supplementary Fig. 4A) showed that *Lcn2* (lipocalin-2) and *Egr2* (early growth response 2) expression decreased in T cell–competent rats after the second PTX cycle compared with the first cycle but remained unchanged in T cell–deficient rats. Consistent with this pattern, *Lcn2* levels decreased in T cell–competent rats compared to -deficient rats after the second cycle. In contrast, *Ephb2* (ephrin receptor B2) expression decreased after the second cycle in T cell–deficient rats but not in T cell–competent rats. *S100b* (S100 calcium-binding protein B) levels were lower in T cell–competent rats than in T cell–deficient rats across both cycles, although both strains showed increased expression after the second PTX cycle. *Aqp1* (aquaporin-1) expression decreased in both strains after the second cycle, whereas *Mfsd2a* (major facilitator superfamily domain-containing protein 2A) expression increased selectively in T cell–deficient rats.

Analysis of anti-inflammatory cytokine genes (Supplementary Fig. 4B) showed that *Il13* (interleukin-13) expression was higher in T cell–deficient rats than in T cell–competent rats after the first PTX cycle but increased in T cell–competent rats after the second cycle, exceeding levels observed in T cell–deficient rats. In contrast, *Il4* (interleukin-4) expression decreased in T cell–competent rats after the second cycle relative to both T cell–deficient rats and the first cycle. Evaluation of proinflammatory cytokine genes (Supplementary Fig. 4C) showed that *Il1β* (interleukin-1 beta) expression remained consistently higher in T cell–deficient rats across both cycles, while *Tnfαip1* (tumor necrosis factor alpha-induced protein 1) expression was similar between strains, with a modest reduction observed in T cell–deficient rats after the second cycle.

These findings demonstrate gene-specific differences in transcriptional programs associated with immune–Schwann cell communication across PTX treatment cycles, with several changes dependent on T cell status.

## 4 Discussion

We investigated the effects of two cycles of PTX treatment and the contribution of T cells in a rat model of PIPN. T cells influenced cold-evoked pain behaviors without substantially affecting the recovery of mechanical hypersensitivity and were associated with differences in anxiety-related exploratory behavior after repeated PTX exposure. Cold allodynia and hyperalgesia developed in T cell–competent rats following the first PTX cycle. Cold allodynia resolved between treatment cycles and re-emerged after the second cycle, whereas cold hyperalgesia persisted throughout the study. In contrast, T cell-deficient rats did not develop significant cold allodynia or hyperalgesia relative to baseline, although withdrawal latencies in the dry-ice test decreased at later time points and approached those of T cell-competent rats. After the second PTX cycle, T cell frequencies were higher in the DRG and sciatic nerve and lower in the spleen. NK cells increased in the sciatic nerve after the second PTX exposure independently of T cells, but increased in the DRG in only T cell–deficient rats, and decreased in the spleen in T cell-competent rats. B cells decreased in the DRG of T cell–deficient rats, while B cells increased in the sciatic nerve of T cell–competent rats after the second cycle of PTX exposure. Macrophage populations showed tissue-specific, T cell–dependent and –independent features, while peripheral gliosis decreased after the second treatment cycle independently of T cell status, accompanied by cell type-specific changes in iNOS-associated signaling. Allodynia, hyperalgesia, and anxiety-like behaviors are well-established features of PIPN in both preclinical and clinical settings (Flatters et al., 2017; Griffiths et al., 2018). However, most preclinical studies rely on a single chemotherapy cycle and do not distinguish between acute and chronic neuropathic phenotypes, limiting translational relevance. In this study, mechanical and cold allodynia resolved between treatment cycles, whereas cold hyperalgesia persisted in T cell-competent rats. Resolution of mechanical allodynia is consistent with prior reports of spontaneous recovery after chemotherapy (Laumet et al., 2019; Sankaranarayanan et al., 2023; Singh et al., 2022), although studies of pain resolution have largely been limited to mechanical endpoints, with little evaluation of cold hypersensitivity.

These findings indicate that mechanical and cold allodynia are transient features of PIPN, whereas cold hyperalgesia persists in T cell-competent rats and anxiety-related exploratory behavior s differs according to T cell status after repeated PTX exposure. The smaller reduction in mechanical withdrawal thresholds observed during the second PTX cycle was accompanied by higher neural T cell frequencies than after the first cycle in T cell–competent rats, consistent with prior reports linking immune cell infiltration to the commencement of pain resolution (Sankaranarayanan et al., 2023; Singh et al., 2022). Consistent with our previous work, T cells transiently influenced the onset of mechanical allodynia during the first cycle (Bakare et al., 2026) but did not substantially affect its development during the second cycle. Our study extends these observations by demonstrating a T cell-dependent pattern of cold allodynia and cold hyperalgesia following PTX treatment. These observations are consistent with a prior report of persistent cold hypersensitivity and intercyclic resolution of mechanical allodynia following repeated paclitaxel treatment (Osborn et al., 2026). Furthermore, the lack of significant cold allodynia or hyperalgesia relative to baseline in T cell–deficient rats is consistent with our previous findings (Bakare et al., 2026) and supports a role for T cells in shaping cold-evoked neuropathic pain responses.

Notably, recovery from mechanical allodynia occurred independently of T cell status, indicating that resolution of tactile hypersensitivity does not require T cell competency in this model. This finding differs from prior studies in immunodeficient mouse models suggesting T cell–mediated resolution of mechanical allodynia (Krukowski et al., 2016; Sankaranarayanan et al., 2023; Singh et al., 2022). This discrepancy may reflect differences in species, sex, and immune context. In particular, previous studies often used female mice with broader immune deficiencies affecting multiple lymphocyte populations (Krukowski et al., 2016; Laumet et al., 2019; Singh et al., 2022; Sorge et al., 2015), whereas we used male rats that are profoundly deficient in T cells but retain functional B and NK cell populations. Collectively, these findings indicate that T cell contributions to PIPN development and recovery are modality- and context-dependent and may differ under conditions of repeated PTX exposure in male rats.

Macrophage polarization plays a critical role in the maintenance and resolution of PIPN (Kavelaars and Cobi, 2021; Kavelaars and Heijnen, 2021). Proinflammatory M1 macrophages promote neuroinflammatory signaling in peripheral nerves and contribute to neuronal hypersensitivity (Chen et al., 2020; Fumagalli et al., 2020; Kalynovska et al., 2020). Consistent with our prior work in which PTX induced a shift toward a proinflammatory macrophage profile independent of T cell status (Bakare et al., 2026), our current study showed that overall M2-like macrophage abundance in peripheral neural tissues was not substantially influenced by T cells across treatment cycles. However, analysis of macrophage subsets revealed more nuanced, tissue-dependent effects.

In the sciatic nerve, T cell–deficient rats exhibited increased CD68^+^CD206^+^ M2-like macrophages after the second PTX cycle, whereas in the DRG, these macrophages increased independently of T cell status. In contrast, systemic M2-like macrophages were reduced after the second PTX cycle regardless of T cell status, indicating a shift in systemic macrophage composition that does not directly parallel changes in peripheral neural tissues. Given that M2 macrophages are associated with anti-inflammatory and pro-regenerative responses linked to pain resolution (Wu et al., 2025), these changes suggest localized, tissue-dependent shifts in macrophage populations rather than uniform changes in overall macrophage abundance.

CD86^+^ M1-like macrophage responses varied by tissue and T cell status. CD11b/c^+^CD163^−^CD86^+^ macrophages increased in the sciatic nerve and spleen of T cell–competent rats, whereas reductions were observed in the DRG of T cell–competent rats and spleen of T cell–deficient rats after the second cycle. Complementary analyses of macrophage activation states demonstrated strain-dependent differences in CD68^+^iNOS^+^ macrophage populations in the DRG, but not in the sciatic nerve. In the sciatic nerve, iNOS-expressing Schwann cells (S100β^+^iNOS^+^) differed between T cell–competent and T cell–deficient rats across both cycles and were further reduced after the second PTX cycle in T cell–competent rats. Because iNOS-associated signaling is linked to enhanced neuroinflammation and neuronal sensitization (Cuhadar et al., 2019; Wu et al., 2025), these region- and cell type–specific differences may contribute to the local inflammatory environment.

The role of B cells in PIPN remains poorly defined, although neuropathic and autoimmune inflammatory pain models, and patient passive transfer studies, suggest that B cell-derived antibodies can promote pain hypersensitivity, through activation of Fcγ receptors and binding to satellite glial cells within DRGs (Cuhadar et al., 2019; Goebel et al., 2021; Guo et al., 2023; Lacagnina et al., 2024). Our previous findings showed that PTX reduces B cell populations in a tissue-specific manner, with some effects differing by T cell status (Bakare et al., 2026). In the present study, B cell populations showed tissue- and T cell–dependent differences across PTX treatment cycles, with increased B cell frequency in the sciatic nerve of T cell–competent rats after the second cycle, reduced B cell frequency in the DRG of T cell–deficient rats, and no change across cycles in the spleen, where B cell levels were consistently higher in T cell–deficient rats. Regulatory B cells have been reported to produce anti-inflammatory cytokines, including IL-10 and TGF-β, which can facilitate resolution of inflammatory pain (Bakare et al., 2026; Pennati et al., 2016), raising the possibility that local B cell accumulation in peripheral nerves reflects a context-dependent immunoregulatory response. In contrast, systemic B cell populations remained elevated in T cell–deficient rats, suggesting differences in B cell distribution across tissues in the absence of T cells. Overall, these findings indicate that tissue- and T cell-dependent differences in B cell populations across PTX treatment cycles.

NK cells have been implicated in the resolution of neuropathic pain. We previously reported that reduced NK cell frequency in peripheral nerves of T cell–deficient rats was associated with the development and persistence of PIPN (Bakare et al., 2026). Consistent with this finding, NK cell frequency was decreased in T cell–deficient rats after the first PTX cycle but increased to levels comparable to T cell–competent rats after the second cycle, indicating dynamic regulation across treatment cycles in the absence of T cells. Increased NK cell activity has been associated with reduced mechanical pain sensitivity and diminished hypersensitivity (Davies et al., 2019; Lassen et al., 2021), consistent with a role for NK cells in modulating pain responses. Notably, mature CD11b/c^+^ NK cells increased during the second PIPN cycle, suggesting that NK cell maturation may be associated with these changes. Tissue specificity was also evident, as NK cell frequency increased in the sciatic nerve after the second PTX cycle irrespective of T cell status, suggesting local NK cell accumulation in peripheral nerves.

T cells have also been implicated in pain modulation, although their effects appear subset- and context-dependent. Some studies have demonstrated protective roles, particularly in female models (Krukowski et al., 2016; Laumet et al., 2019; Sankaranarayanan et al., 2023; Singh et al., 2022), but others have reported that functional subsets such as Th1 and Th17 CD4^+^ T cells exacerbate hyperalgesia (Davoli-Ferreira et al., 2020; Kavelaars and Heijnen, 2021; Kleinschnitz et al., 2006; Moalem et al., 2004). Conversely, Th2 and regulatory T cells (Tregs) have been shown to have analgesic, anti-inflammatory, and restorative effects in nerve injury models of neuropathic pain (Austin et al., 2012; Fiore et al., 2023; Mendoza et al., 2025; Midavaine et al., 2025; Moalem et al., 2004). In a range of neuropathic and primary chronic pain conditions, there is strong evidence for increased systemic memory T cell abundance and activation, altered Th1 and Th17 subsets, and increased analgesic and tissue-restorative Tregs, presumably in response to an ongoing state (Heyn et al., 2019; Kogias et al., 2025; Luchting et al., 2015; O'Brien et al., 2021; Russo et al., 2019).

Our previous work showed that PTX reduced T cell infiltration in peripheral nerves, thereby contributing to mechanical and cold hypersensitivity (Bakare et al., 2026). In the current study, T cell frequencies were higher after the second PTX cycle in both the DRG and sciatic nerve and lower in the spleen than after the first cycle, indicating differential regulation across neural and systemic compartments. In the DRG and sciatic nerve, this increase was accompanied by reduced CD4^+^ and increased single-positive CD8^+^ T cell frequencies, resulting in a lower CD4^+^/CD8^+^ ratio. In contrast, CD4^+^ and CD8^+^ T cell frequencies in the spleen did not differ significantly across cycles. It is possible that these changes in CD4^+^ and CD8^+^ T cell populations contribute to altered cold sensitivity following repeated PTX exposure. Changes in CD4^+^ T cell populations, including potentially antinociceptive Tregs, may represent one mechanism underlying this response (Sakaguchi et al., 2010). We also observed differences in anxiety-related exploratory behavior between T cell-competent and T cell-deficient rats after the second PTX cycle. T cell subsets have been implicated in affective behaviors, with Th1 cells associated with anxiety- and depressive-like behaviors and Tregs with protective effects in preclinical models (Kim et al., 2011, 2012). However, functional T cell subsets were not characterized in the present study, precluding attribution of these behavioral findings to a specific T cell population. These findings indicate that T cell responses to repeated PTX exposure are tissue- and subset-specific, with potential implications for local neuroimmune regulation and affective behavior during PIPN. Future investigation of functional subsets is warranted to define their contribution to mechanical and cold hypersensitivity and anxiety-related behavior.

PIPN is associated with neuroinflammation characterized by gliosis in peripheral neural tissues (Fumagalli et al., 2020; Sankaranarayanan et al., 2023). Activation of satellite glial cells in the DRG and Schwann cells in the sciatic nerve can alter neuronal excitability and promote inflammatory pain states (Fumagalli et al., 2020; Luo et al., 2025; Sankaranarayanan et al., 2023). Consistent with prior reports, satellite glial activation in the DRG was evident after the first PTX treatment cycle. GS^+^ satellite glial cells were reduced after the second PTX cycle in both T cell–competent and -deficient rats, indicating a reduction that was not dependent on T cells. In contrast, iNOS associated signaling showed more specific, T cell–dependent effects. T cell–competent rats exhibited greater changes in iNOS^+^ cell frequencies across treatment cycles in the DRG, sciatic nerve, and Schwann cell populations than T cell–deficient rats. These findings indicate that repeated PTX exposure is associated with reduced satellite glial activation alongside more selective, T cell–dependent modulation of inflammatory signaling within peripheral neural tissues.

Gene-specific transcriptional changes after repeated PTX exposure further supported T cell–dependent modulation of neuroinflammatory signaling in the sciatic nerve. Rather than a uniform shift in inflammatory gene expression, these changes were gene-specific and differed by T cell status, consistent with distinct immune-glial signaling programs. In T cell–competent rats, reductions in *Lcn2* and *Egr2* expression after the second cycle suggest altered Schwann cell–associated signaling (Jeon et al., 2013; Jessen and Mirsky, 2008; Jha et al., 2014; Tammia et al., 2018), whereas T cell–deficient rats exhibited a distinct pattern characterized by changes in *Ephb2* and *Mfsd2a*, implicating alternative pathways related to Schwann cell function and blood–nerve barrier regulation (Reinhold et al., 2023). S100b expression was consistently lower in T cell–competent rats but increased after repeated PTX exposure in both groups, indicating context-dependent regulation of Schwann cell–associated signaling. Cytokine expression patterns further support this divergence, with dynamic, cycle-dependent changes in IL-13 (Singh et al., 2022) and persistent elevation of IL-1β in T cell–deficient rats, potentially in association with higher S100b expression (Niven et al., 2015). TNF-αip1 expression showed only modest changes, with a reduction in T cell–deficient rats after the second cycle. Together, these findings indicate that repeated PTX exposure engages distinct, T cell–dependent transcriptional programs that shape immune–Schwann cell interactions, rather than producing a coordinated or uniformly regulated inflammatory response.

A limitation of this study is the lack of sex-specific analyses, as immune mechanisms underlying CIPN/PIPN can differ between males and females, particularly with respect to T cell–mediated effects and pain resolution. The use of a single strain and a single T cell–deficient model may not fully capture the range of immune variability observed clinically. Although RNU rats are widely used as a T cell–deficient model, the Foxn1 mutation causes thymic epithelial abnormalities and may produce broader alterations in immune homeostasis beyond T-cell deficiency. We used heterozygous RNU ^±^ littermates as T cell-competent controls to maintain a matched genetic background; however, differences in thymic architecture and lymphocyte populations between heterozygous and homozygous wild-type animals have been reported. Thus, our findings reflect differences between RNU^−/−^ rats and their RNU ^±^ littermates and should not be interpreted as a direct comparison with homozygous RNU^+/+^ animals. Future studies incorporating complementary models, including homozygous RNU^+/+^ controls, T cell reconstitution, and deeper T cell subset phenotyping, may help further define these relationships. Finally, behavioral follow-up after the second treatment cycle ended before mechanical withdrawal thresholds returned to baseline, limiting our ability to distinguish a reduction in the magnitude of mechanical hypersensitivity from a change in its time course. Longer-term studies will be needed to characterize the trajectory of pain resolution or persistence following repeated PTX exposure.

In conclusion, repeated PTX exposure differentially modulates behavioral and neuroimmune features of PIPN. Mechanical allodynia represents a transient phenotype that resolves between treatment cycles, with a smaller reduction in mechanical withdrawal thresholds observed during the measured period following the second cycle. In contrast, cold hypersensitivity differs according to T cell status, with persistent cold hyperalgesia and transient cold allodynia observed in T cell-competent rats but not reaching significance relative to baseline in T cell-deficient rats. Across these phases, PTX exposure is associated with dynamic, tissue-specific changes in immune cell populations, including T cells, NK cells, macrophages, and B cells, without a uniform pattern of inflammatory activation. Instead, neuroimmune responses shift from broader glial activation after the first cycle to more selective, cell type– and gene-specific regulation with repeated exposure. These changes include T cell–dependent modulation of macrophage populations and activation states, iNOS-associated signaling, and transcriptional programs within Schwann cells and peripheral nerves. Overall, repeated PTX exposure engages context-dependent neuroimmune mechanisms associated with distinct neuropathic pain phenotypes and their persistence, rather than a single coordinated inflammatory pathway.

## CRediT authorship contribution statement

**Ahmed Olalekan Bakare:** Writing – review & editing, Writing – original draft, Visualization, Validation, Methodology, Investigation, Formal analysis, Data curation, Conceptualization. **Paul J. Austin:** Writing – review & editing, Validation. **Michele Curatolo:** Writing – review & editing, Validation. **Kimberly E. Stephens:** Writing – review & editing, Validation. **Eellan Sivanesan:** Writing – review & editing, Writing – original draft, Visualization, Validation, Supervision, Resources, Project administration, Methodology, Investigation, Funding acquisition, Formal analysis, Data curation, Conceptualization.

## Ethics declaration

This study was approved by the Johns Hopkins University Animal Care and Use Committee (ACUC). (Approval No. RA23M161). This study was conducted in accordance with the ARRIVE (Animal Research: Reporting of In Vivo Experiments) guidelines as well as the following guidelines for animal welfare and/or reporting: Guide for the Care and Use of Laboratory Animals, 8th Edition.

## Funding

This study was conducted at the Johns Hopkins University and supported by grant CA255428 from The National Cancer Institute–National Institutes of Health (Bethesda, MD, USA) and by funding from The Department of Anesthesiology and Critical Care Medicine, Johns Hopkins University School of Medicine (E.S.). Funders had no role in study design, data collection, or data interpretation, or in the decision to submit the work for publication.

## Declaration of competing interest

The authors declare the following financial interests/personal relationships which may be considered as potential competing interests:Michele Curatolo reports a relationship with 4E Therapeutics that includes: employment and equity or stocks. If there are other authors, they declare that they have no known competing financial interests or personal relationships that could have appeared to influence the work reported in this paper.

## Data Availability

Data will be made available on request.
