# Supplementary material for: T cell–dependent modulation of pain behaviors in a rat model of recurring paclitaxel-induced peripheral neuropathy
Source: Brain Behav Immun Health. 2026 Sep 8;57:101349. doi: 10.1016/j.bbih.2026.101349 (PMC13587728; doi:10.1016/j.bbih.2026.101349)
Supplement: Supplementary file 1 [file mmc1.docx]

**Supplementary Material**

**Supplementary Methods**

**Flow cytometry:** Immune cell populations, including T cells, B cells, natural killer (NK) cells, and macrophages, in the dorsal root ganglia (DRG; L4–L6), distal sciatic nerve, and spleen were analyzed by flow cytometry. After being anesthetized with isoflurane, rats were transcardially perfused with phosphate-buffered saline (PBS) to remove circulating blood cells. Tissues were rapidly dissected on ice and processed into single-cell suspensions. Each tissue sample was digested in enzyme buffer (HBSS containing 10 μM HEPES, 5 mg/mL bovine serum albumin [BSA], 1.6 mg/mL collagenase IV, and 100 μg/mL DNase I) at 37°C for 30 minutes with gentle agitation. Digestion was terminated by adding 2 mL fluorescence-activated cell sorting (FACS) buffer (PBS with 10% heat-inactivated fetal bovine serum [FBS] and 1 mM EDTA), and the suspension was passed through a 70 μm cell strainer. The cell suspension was washed with an additional 8 mL of FACS buffer, centrifuged at 400 × *g* for 15 minutes at 4°C, and resuspended in 1 mL of FACS buffer. Spleen samples were treated with ammonium–chloride–potassium lysis buffer (Quality Biological, cat. #118-156-101, Gaithersburg, MD, USA) to remove red blood cells and then filtered through a 40 μm strainer.

Cells were stained with a live/dead fixable aqua dead cell stain (Thermo Fisher Scientific, cat. #34957) for 30 minutes at room temperature, followed by Fc receptor blocking with anti-rat CD32. Subsequently, 100 μL aliquots of each sample were incubated for 30 minutes with the following mouse anti-rat antibodies: CD45 (BD Biosciences, cat. #567734, clone OX-1), CD11b/c (BD Biosciences, cat. #746165, clone OX-42), CD163 (Abcam, cat. #MCA342B, clone ED2; secondary BV711 streptavidin, BD Biosciences, cat. #563262), CD86 (BD Biosciences, cat. #743211, clone 24F), CD4 (BD Biosciences, cat. #740723, clone OX-35), CD8a (BD Biosciences, cat. #740041, clone OX-8), and a T/B/NK cell cocktail (BD Biosciences, cat. #558495; CD3, clone IF4; CD45RA, clone OX-33; CD161a, clone 10/78). Two staining panels were used:

• **Panel 1:** CD45, CD11b/c, T/B/NK cell cocktail, CD4, and CD8a

• **Panel 2:** CD45, T/B/NK cell cocktail, CD11b/c, CD86, and CD163

After being stained, cells were washed, resuspended in FACS buffer, and analyzed on a BD Symphony A3 flow cytometer at the Johns Hopkins University Bloomberg Flow Cytometry and Immunology Core Facility. Data were analyzed with FlowJo software (version 10.10.0, BD Biosciences).

**Immunohistochemistry:** Rats were deeply anesthetized and perfused transcardially with PBS followed by 4% paraformaldehyde (PFA) in PBS. The distal segment (1 cm) of the sciatic nerve, immediately proximal to the trifurcation, and the L4–L6 DRG were rapidly collected into cold 4% PFA and post-fixed at 4°C for 24 hours. Tissues were then transferred to 30% sucrose solution for cryoprotection and incubated for 48–72 hours at 4°C. Samples were embedded in paraffin, sectioned at 10 μm thickness on a microtome, and mounted on glass slides for immunohistochemical staining, as described previously (Bakare et al., 2026).

Following deparaffinization, clearing, rehydration, antigen retrieval, and blocking, tissue sections were incubated overnight at 4°C with primary antibodies under gentle agitation. Sections were then washed and incubated with appropriate secondary antibodies for 2 hours at room temperature. Fluorescent images were acquired on a Leica SP8 confocal microscope equipped with Leica Application Suite X software (version 3.5.7) under a 20× objective lens. QuPath software (version 0.5.1) was used for quantitative analysis of immunoreactive cells. The list of primary and secondary antibodies used is provided in Supplementary Table 1.

**Quantitative real-time PCR (qPCR) analysis.** Distal sciatic nerve tissues were harvested from T cell–competent and T cell–deficient rats and immediately preserved in RNAlater solution (Thermo Fisher Scientific) to stabilize RNA. Samples were homogenized in TRIzol reagent (Sigma-Aldrich, Italy) with the Bio-Rad ReadyPrep™ Mini Grinders Kit (#1632146) according to the manufacturer’s instructions. The samples were sonicated in three 10-second pulses to ensure complete lysis. Total RNA was extracted by using the RNeasy® Mini Kit (Qiagen, #74104) according to the manufacturer’s protocol and quantified in a NanoDrop spectrophotometer. Extracted RNA was stored at −80°C until use.

First-strand complementary DNA (cDNA) was synthesized by using the SuperScript™ IV VILO™ Master Mix (Invitrogen, Thermo Fisher Scientific, #11766050) according to the manufacturer’s protocol. Quantitative PCR was performed on a 7900HT Fast Real-Time PCR System (Applied Biosystems, Waltham, MA, USA) in PowerUp™ SYBR™ Green Master Mix (Thermo Fisher Scientific, #A25742). Each reaction contained 200 nM forward and reverse primers and cDNA template.

Relative gene expression was calculated by using the 2-ΔΔCT method, with normalization to the housekeeping gene *GAPDH*. Primer sequences are provided in Supplementary Table 2.

**Supplementary Figure 1. Flow cytometry gating strategies for immune cell analysis in DRG, sciatic nerve, and spleen.** (A) Gating strategy for immune cell identification in the DRG and sciatic nerve. (B) Gating strategy for the spleen. (C) Fluorescence-minus-one (FMO) controls for antibodies used to define positive gating thresholds. DRG, dorsal root ganglia.

**Supplementary Figure 2. CD68⁺CD206⁺ M2-like macrophages differ across PTX treatment cycles in the DRG and sciatic nerve.** (A) Representative confocal images and quantification of CD68⁺CD206⁺ M2-like macrophages in DRG of T cell-competent and T cell-deficient rats. CD68⁺CD206⁺ M2-like macrophage frequencies were higher after the second PTX cycle than after the first in the DRG, independent of T cell status. (B) Representative confocal images and quantification of CD68⁺CD206⁺ M2-like macrophages in sciatic nerve. CD68⁺CD206⁺ M2-like macrophage frequencies were higher after the second PTX cycle than after the first in T cell-deficient rats. Data are presented as mean + SEM. One-way ANOVA with Tukey’s post hoc test. *p<0.05, **p<0.01, ***p<0.001. N = 3 per group. DRG, dorsal root ganglia; PTX, paclitaxel; RNU+/−, T cell-competent; RNU−/−, T cell-deficient.

**Supplementary Figure 3. NK cell subsets differ across PTX treatment cycles in the DRG, sciatic nerve, and spleen. (A)** Representative flow cytometry plots and quantification of CD11b/c⁺ (mature) and CD11b/c⁻ (immature) NK cells (live CD45⁺) in the DRG. After the second PTX cycle, CD11b/c⁺ NK cell frequencies were higher than after the first cycle in both the DRG and sciatic nerve, independent of T cell status. CD11b/c⁻ NK cells were higher after the second cycle in the DRG regardless of genotype and in the sciatic nerve only in T cell–competent rats. **(B)** Representative plots and quantification of CD11b/c⁺ and CD11b/c⁻ NK cells in spleen. Neither genotype showed differences in NK cell subset frequencies between PTX treatment cycles. NK cell subsets were quantified within the total live CD45⁺CD161a⁺ cell population. Data are presented as mean + SEM. One-way ANOVA with Tukey’s post hoc test **p*<0.05, ***p*<0.01, ****p*<0.001. N   = 3 per group. DRG, dorsal root ganglia; NK, natural killer; PTX, paclitaxel; RNU^+/-^, T cell–competent; RNU^-/-^, T cell–deficient.

**Supplementary Figure 4. Gene expression in the sciatic nerve differs across PTX treatment cycles in a T cell–dependent manner.** (A) Relative expression of genes associated with proinflammatory signaling and Schwann cell–related function after the first and second PTX cycles. After the second cycle, LCN2 and EGR2 expression decreased in T cell–competent rats, whereas EphB2 expression decreased in T cell–deficient rats. S100B expression increased and Aqp1 expression decreased independent of T cell status. (B) Relative expression of anti-inflammatory cytokine genes. IL-13 expression increased and IL-4 expression decreased in T cell–competent rats after the second cycle, but neither changed in T cell–deficient rats. (C) Relative expression of proinflammatory cytokine genes. IL-1β expression did not differ between cycles or strains, whereas Tnfαip1 expression was reduced in T cell–deficient rats after the second cycle. (D) STRING-derived gene–gene interaction network illustrating potential interactions between immune cytokines and Schwann cell-associated genes. IL-1β is positioned as a central node linking immunoregulatory cytokines associated with macrophage polarization with Schwann cell-related genes, including S100B, LCN2, and EGR2, suggesting coordinated inflammatory and glial responses across PTX treatment cycles. Data are presented as mean + SEM. One-way ANOVA with Tukey’s post hoc test **p*<0.05, ***p*<0.01, ****p*<0.001, *****p*<0.0001. N = 3 per group. Aqp1, aquaporin-1; EGR2, early growth response 2; Ephb2, ephrin receptor B2; IL, interleukin; Lcn2, lipocalin-2; Mfsd2a, major facilitator superfamily domain-containing protein 2A; PTX, paclitaxel; RNU^+/-^; T cell–competent; RNU^-/-^, T cell–deficient; S100b, S100 calcium-binding protein B; TNFAIP1, tumor necrosis factor alpha-induced protein 1.

**Supplementary Table 1: Detailed description of all antibodies**

| **Antibody/Stain** | **Vendor** | **Clone** | **Catalogue #** | **Dilution** |
| --- | --- | --- | --- | --- |
| live/dead fixable aqua dead cell stain | ThermoFisher Scientific |  | 34957 | 1 µL:1x10^6^ |
| CD45 | BD Bioscience | OX-1 | BD567734 | 3 µL:1x10^6^ |
| CD11b/c | BD Bioscience | OX-42 | BD746165 | 3 µL:1x10^6^ |
| CD163 | Abcam | ED-2 | MCA342B | 2.5 µL:1x10^6^ |
| CD86 | BD Bioscience | 24F | BD743211 | 1 µL:1x10^6^ |
| CD4 | BD Bioscience | OX-35 | BD740723 | 2 µL:1x10^6^ |
| CD8a | BD Bioscience | OX-8 | BD740041 | 2 µL:1x10^6^ |
| T/B/NK cell cocktail | BD Bioscience | CD3: clone IF4, CD45RA: clone OX-33, CD161a: clone 10/78 | BD558495 | 10 µL:1x10^6^ |
| BV711 streptavidin | BD Bioscience | 563262 | 563262 | 2 µL:1x10^6^ |
| Antimouse CD68 | Bio-Rad |  | MCA341R | 1:100 µL |
| Antimouse S100B | Sigma |  | S2532-2ml | 1:100 µL |
| Antirabbit CD206 | Abcam |  | AB64693 | 1:500 µL |
| Antirabbit iNOS |  |  | NBPI-33780 | 1:100 µL |
| Antirabbit glutamine synthetase | Abcam |  | AB176562 | 1:1000 µL |
| Alexa Fluor 488 | Invitrogen |  | A11001 | 1:1000 µL |
| Alexa Fluor 568 | Invitrogen |  | A11036 | 1:1000 µL |
| Alexa Fluor 647 | Invitrogen |  | A-21245 | 1:1000 µL |
| DAPI | Invitrogen |  | AS-83210 | 1:1000 µL |

**Supplementary Table 2: Detailed description of primers used for qPCR**

| **Gene** | **Description** | **Sequence** |
| --- | --- | --- |
| Gapdh | Forward | GGCAAGTTCAACGGCACAG |
|  | Reverse | CGCCAGTAGACTCCACGAC |
| Lcn2 | Forward | AACGTCACTTCCATCCTCGTC |
|  | Reverse | AATCGCTCCTTCAGTTCATCG |
| Ephb2 | Forward | ACCTCAGTTCGCCTCTGTGAA |
|  | Reverse | GGCTCACCTGGTGCATGAT |
| S100b | Forward | TACCCTCCTAGTCCTCGGACAC |
|  | Reverse | AGCTTGTGCTTGTCACCCTCT |
| Aqp1 | Forward | TGTGCTCACCCGCAACTTCT |
|  | Reverse | GCCACTGGTCCACACCTTCAT |
| Mfsd2a | Forward | CATCAGTACCGAGCAGAGTGAG |
|  | Reverse | GGTACGATTGACAACTTCTGAGG |
| Egr2 | Forward | GGCCGTAGACAAAATCCCAGT |
|  | Reverse | GGCCTCTTCTCTCCGGTCAT |
| Il13 | Forward | AAGACCAGAAGACTTCCC |
|  | Reverse | AGGCCATTCAATATCCTCTG |
| Il1β | Forward | TAAGCCAACAAGTGGTATTC |
|  | Reverse | AGGTATAGATTCTTCCCCTTG |
| Il4 | Forward | GAACCAGGTCACAGAAAAAG |
|  | Reverse | GGGAAGTAAAATTTGCGAAG |
| Tnfaip1 | Forward | AAAATCTATTCTGCCTTGACC |
|  | Reverse | GATCCTCCTGTCTTATCCTG |

**Supplementary Table 3. Effects of repeated PTX exposure on pain-like behavioral responses of T cell–competent and T cell–deficient rats**

| **Parameter** | **T cell–competent rats** | | | **T cell–deficient rats** | | |
| --- | --- | --- | --- | --- | --- | --- |
|  | **Post-cycle 1**  **(wk 4)** | **Pre-cycle 2**  **(wk 10)** | **Post-cycle 2**  **(wk 14)** | **Post-cycle 1**  **(wk 4)** | **Pre-cycle 2**  **(wk 10)** | **Post-cycle 2**  **(wk 14)** |
| **Mechanical allodynia** | Very high | Completely resolved | Moderate | Very high | Completely resolved | High |
| **Cold allodynia** | Very high | Completely resolved | Moderate | Absent | Absent | Absent |
| **Cold hyperalgesia** | Very high | Very high | Very high | Absent | Absent | Absent |
| **Anxiety-like behavior** | High | Very high | Moderate | High | Very high | Very high |

** Qualitative descriptors reflect the magnitude and temporal pattern of behavioral responses relative to baseline and between treatment cycles.*

**Supplementary Table 4. Summary of flow cytometric immune cell changes across tissues, treatment cycles, and T cell status**

|  | **T cell–competent rats** | **T cell–deficient rats** | **Post-cycle 1**  **(wk 4)** | **Post-cycle 2**  **(wk 14)** |
| --- | --- | --- | --- | --- |
|  | **Post-cycle 1**  **(wk 4)**  **VS**  **Post-cycle 2**  **(wk 14)** | **Post-cycle 1**  **(wk 4)**  **VS**  **Post-cycle 2**  **(wk 14)** | **T cell–competent rats**  **VS**  **T cell–deficient rats** | **T cell–competent rats**  **VS**  **T cell–deficient rats** |
| **DRG** |  |  |  |  |
| M2-like macrophages | No difference  (NS) | No difference (NS) | No difference (NS) | No difference  (NS) |
| M1-like macrophages | Decreased  (*) | No difference (NS) | Decreased  (**) | Decreased  (*) |
| B cells | No difference (NS) | Decreased  (***) | Increased  (*) | No difference  (NS) |
| NK cells | No difference (NS) | Increased  (***) | Increased  (*) | No difference  (NS) |
| **Sciatic nerve** |  |  |  |  |
| M2-like macrophages | No difference (NS) | No difference (NS) | No difference (NS) | No difference  (NS) |
| M1-like macrophages | Increased  (*) | No difference (NS) | Increased  (*) | Decreased  (**) |
| B cells | Increased  (*) | No difference (NS) | No difference (NS) | No difference  (NS) |
| NK cells | Increased  (**) | Increased  (**) | No difference (NS) | No difference  (NS) |
| **Spleen** |  |  |  |  |
| M2-like macrophages | Decreased  (*) | Decreased  (***) | Increased  (*) | No difference  (NS) |
| M1-like macrophages | No difference (NS) | Decreased  (*) | No difference (NS) | Decreased  (**) |
| B cells | No difference (NS) | No difference (NS) | Increased  (***) | Increased  (**) |
| NK cells | Decreased  (*) | No difference  (NS) | No difference (NS) | Increased  (*) |

** p < 0.05, ** p < 0.01, *** p < 0.001*

**Reference**

Bakare, A.O., Limerick, G., Goel, V., Banik, R.K., Zheng, L., Shepherd, A.J., Glunde, K., Zheng, Q., Sivanesan, E., 2026. T cells modulate the development and maintenance of painful paclitaxel-induced peripheral neuropathy in RNU rats. Mol Pain 22, 17448069261418431.
